# Suppression of Inflammatory Cardiac Cytokine Network in Rats with Untreated Obesity and Pre-Diabetes by AT2 Receptor Agonist NP-6A4

**DOI:** 10.3389/fphar.2021.693167

**Published:** 2021-06-18

**Authors:** Madhavi P. Gavini, Abuzar Mahmood, Anthony M. Belenchia, Paige Beauparlant, Senthil A. Kumar, Sivakumar Ardhanari, Vincent G. DeMarco, Lakshmi Pulakat

**Affiliations:** ^1^ Novopyxis Inc., Boston, MA, United States; ^2^ Dalton Cardiovascular Research Center, Columbia, MO, United States; ^3^ Department of Medicine, Boston, MA, United States; ^4^ Harry S. Truman Memorial VA Hospital, Columbia, MO, United States; ^5^ Department of Nutrition and Exercise Physiology, University of Missouri, Columbia, MO, United States; ^6^ Tufts Medical Center and Department of Medicine, Molecular Cardiology Research Institute, Tufts University School of Medicine, Boston, MA, United States

**Keywords:** obesity, cardiac dysfunction, inflammatory cytokines, myocardial strain, AT2 receptor, heart disease, NP-6A4

## Abstract

Obesity affects over 42% of the United States population and exacerbates heart disease, the leading cause of death in men and women. Obesity also increases pro-inflammatory cytokines that cause chronic tissue damage to vital organs. The standard-of-care does not sufficiently attenuate these inflammatory sequelae. Angiotensin II receptor AT2R is an anti-inflammatory and cardiovascular protective molecule; however, AT2R agonists are not used in the clinic to treat heart disease. NP-6A4 is a new AT2R peptide agonist with an FDA orphan drug designation for pediatric cardiomyopathy. NP-6A4 increases AT2R expression (mRNA and protein) and nitric oxide generation in human cardiovascular cells. AT2R-antagonist PD123319 and AT2RSiRNA suppress NP-6A4-effects indicating that NP-6A4 acts through AT2R. To determine whether NP-6A4 would mitigate cardiac damage from chronic inflammation induced by untreated obesity, we investigated the effects of 2-weeks NP-6A4 treatment (1.8 mg/kg delivered subcutaneously) on cardiac pathology of male Zucker obese (ZO) rats that display obesity, pre-diabetes and cardiac dysfunction. NP-6A4 attenuated cardiac diastolic and systolic dysfunction, cardiac fibrosis and cardiomyocyte hypertrophy, but increased myocardial capillary density. NP-6A4 treatment suppressed tubulointerstitial injury marker urinary β-NAG, and liver injury marker alkaline phosphatase in serum. These protective effects of NP-6A4 occurred in the presence of obesity, hyperinsulinemia, hyperglycemia, and hyperlipidemia, and without modulating blood pressure. NP-6A4 increased expression of AT2R (consistent with human cells) and cardioprotective erythropoietin (EPO) and Notch1 in ZO rat heart, but suppressed nineteen inflammatory cytokines. Cardiac miRNA profiling and *in silico* analysis showed that NP-6A4 activated a unique miRNA network that may regulate expression of AT2R, EPO, Notch1 and inflammatory cytokines, and mitigate cardiac pathology. Seventeen pro-inflammatory and pro-fibrotic cytokines that increase during lethal cytokine storms caused by infections such as COVID-19 were among the cytokines suppressed by NP-6A4 treatment in ZO rat heart. Thus, NP-6A4 activates a novel anti-inflammatory network comprised of 21 proteins in the heart that was not reported previously. Since NP-6A4’s unique mode of action suppresses pro-inflammatory cytokine network and attenuates myocardial damage, it can be an ideal adjuvant drug with other anti-glycemic, anti-hypertensive, standard-of-care drugs to protect the heart tissues from pro-inflammatory and pro-fibrotic cytokine attack induced by obesity.

## Introduction

Heart disease, the number one killer of men and women, is induced by obesity that affects 42.4% of the United States population (WHO 2020; Virani et al., 2021; Piché et al., 2020). Obesity induces pre-diabetes and cardiometabolic disease *via* increasing the levels of pro-inflammatory and pro-fibrotic cytokines that cause cardiac fibrosis, hypertrophy, reduction in capillary density (capillary rarefaction) and significant myocardial damage (Cavalera et al., 2014; Riaz et al., 2018; Strain and Paldánius, 2018; CDC: Centers for Disease Control and Prevention, 2020; CDC: Centers for Disease Control and Prevention, 2021). Obesity also increases the severity and lethality of infections by exacerbating the underlying cardiac damage (Mauvais-Jarvis., 2020; Tartof et al., 2020). Conventional cardio-protective and anti-inflammatory drugs are insufficient to attenuate this inflammatory onslaught and subsequent myocardial structural damage in obese patients. Thus, there is a critical need for new treatment paradigms to protect the heart from obesity-induced cardiometabolic disease (Fang et al., 2017; Levick and Widiapradja, 2020). Angiotensin II (Ang II) receptor (AT2R) is an anti-inflammatory molecule and a promising target for therapeutic intervention (Chow et al., 2016; Kaschina et al., 2017; Bennion et al., 2018). Transgenic overexpression of AT2R in murine models is cardioprotective, and its deficiency is implicated in myocardial damage in obese and diabetic rats and humans (Altarche-Xifró et al., 2009; Tousoulis et al., 2010; Qi et al., 2012; Chow et al., 2016; Lum-Naihe et al., 2017). There are no AT2R agonists in clinic for heart disease. We and others have shown that AT2R activates reparative signaling and attenuates inflammatory signaling by the AT1R subtype that induces hypertension, fibrosis and cardiac structural damage (Kumar et al., 2002; Kurisu et al., 2003). Therefore, we hypothesized that increasing the expression and anti-inflammatory signaling by AT2R would mitigate pathologic cardiac remodeling and cardiac dysfunction caused by obesity-induced increases in pro-inflammatory and pro-fibrotic cytokines.

**FIGURE 1:**
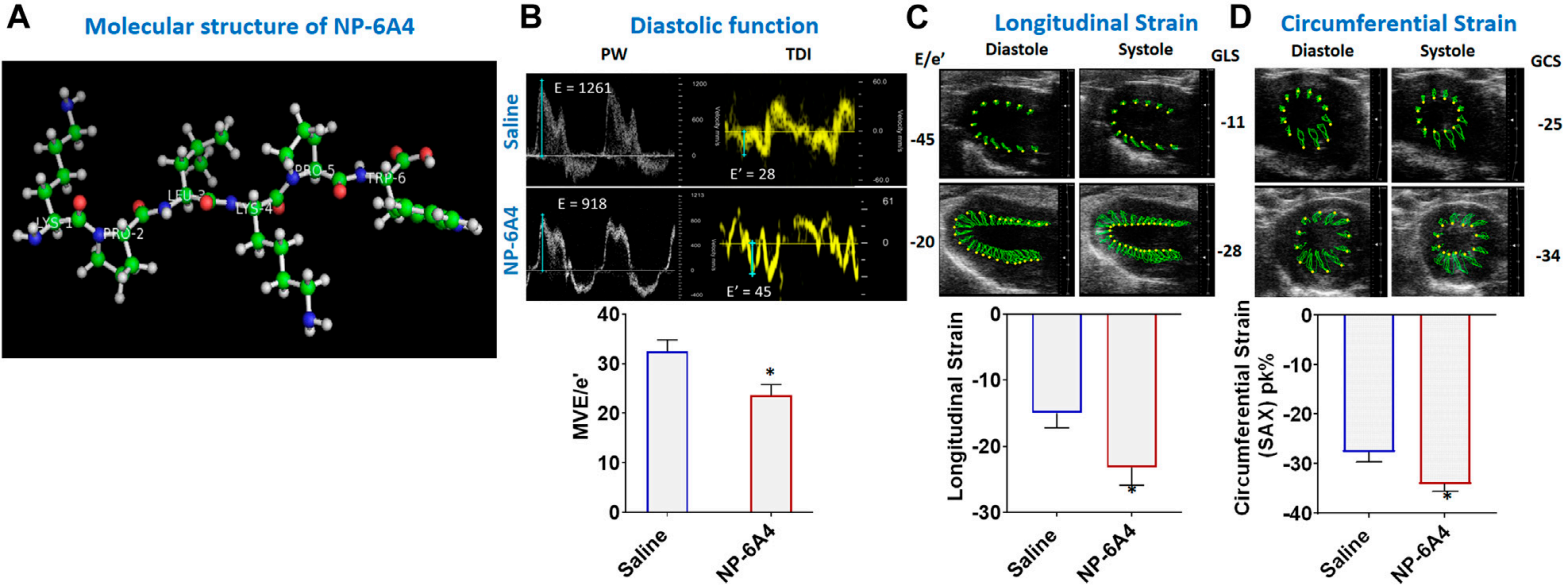
NP-6A4 attenuates cardiac diastolic dysfunction and improves myocardial longitudinal and circumferential strain in ZO rats with untreated diabetes and obesity. **(A)** Structure of NP-6A4, a patented six amino acid peptide agonist of the AT2R with the orphan drug designation for pediatric cardiomyopathy from the FDA. **(B)** Representative Pulse Wave Doppler and Tissue Doppler spectra from a saline- **(top panels)** or NP-64A-treated **(bottom panels)** ZO rat. Note the lower E/e’ ratio in the NP-64A4 treated rat compared to the untreated rat, indicating lower LV filling pressure with NP-64A treatment. **(C)** Representative images taken in the parasternal long axis view from a saline-treated rat **(top panes)** taken at end-diastole and end-systole indicate endocardial longitudinal deformation during the cardiac cycle compared to those from an NP-64A4-treated rat **(bottom panels)**. **(D)** Representative images taken in the short axis view from a saline-treated rat **(top panels)** taken at end diastole and end systole indicate endocardial circumferential deformation during the cardiac cycle compared to those from an NP-64A4-treated rat **(bottom panels)**.

NP-6A4 is a 768 Da, six amino acid peptide drug (Figure 1A) designed to specifically bind and activate AT2R and has an FDA designation for pediatric cardiomyopathy (FDA US Food and Drug Administration 2017). We showed that NP-6A4 treatment increased survival of mouse cardiomyocyte HL-1 cells, and human coronary artery vascular smooth muscle cells (hCAVSMCs) subjected to acute nutrient stress better than four β1-adrenergic receptor blockers (atenolol, metoprolol, carvedilol and nebivolol) and the AT1R blocker losartan (Mahmood and Pulakat, 2015). We also reported that NP-6A4 increases 1) AT2R expression and signaling in hCAVSMCs and endothelial cells (ECs), 2) cellular respiration of hCAVSMCs, 3) nitric oxide generation by ECs, and 4) reduces reactive oxygen species induced by exposure to Doxorubicin in hCAVSMCs (Toedebusch et al., 2018). The protective effects of NP-6A4 on hCAVSMCs and hCAECs were inhibited by pre-treatment with the AT2R-specific antagonist PD123319 or anti-AT2R siRNA, confirming NP-6A4-effects are mediated through AT2R (Mahmood and Pulakat, 2015; Toedebusch et al., 2018). Moreover, NP-6A4 reduced aortic stiffness and proteolytic activity in a mouse model (Sharma et al., 2020). Since NP-6A4 could increase AT2R expression and signaling, we tested whether NP-6A4 could mitigate pathologic cardiac remodeling and dysfunction caused by a combination of obesity and T2DM in a rat model.

We chose male Zucker obese (ZO) rats (Strain Code 185 from Charles River) that harbor a leptin receptor mutation (fa/fa) that causes hyperphagia and exhibit reduction in capillary density (capillary rarefaction), and cardiac fibrosis as the rat model for this study (Zhou et al., 2010; Frisbee et al., 2014; Luck et al., 2017). By the age of nine weeks, ZO rats exhibit severe obesity and insulin resistance, cardiac dysfunction with preserved ejection fraction, a characteristic cardiac pathology of obese and pre-diabetic individuals (Zhou et al., 2010; Maack et al., 2018). Therefore, male ZO rat is an appropriate pre-clinical model for evaluating the cardioprotective effects of NP-6A4 in conditions of untreated obesity. Supplementary Table S1 confirms these metabolic features of 10-week old male ZO rats compared to non-diabetic lean rats. Since our goal was to test whether NP-6A4 could mitigate cardiac structural and functional damage in the presence of untreated obesity and pre-diabetes, we did not treat ZO rats used in this study with any of the established anti-glycemic, anti-hypertensive or anti-lipidemic drugs. At the age of 11-weeks ZO rats were treated with either saline or NP-6A4 (1.8 mg/kg/day) for a two-week period. To gain mechanistic insight into NP-6A4’s mode of action, we investigated how it modulated cardiac cytokines. Here we report that NP-6A4 attenuated cardiac functional and structural damage in obese and pre-diabetic ZO rats and suppressed a large array of inflammatory cytokines in the heart that are implicated in cytokine storm caused by chronic and acute cardiac inflammation caused by metabolic and infectious diseases.

## Materials and Methods

### Animals, Fasting Plasma Profile, HbA1c, and Urine Chemistry

All animal procedures used in this study were approved prior to the beginning of these studies by the Harry S. Truman Memorial Veterans Hospital (HSTMVH) Subcommittee for Animal Safety and University of Missouri IACUC. All animals were cared for in accordance with the Guidelines for the Care and Use of Laboratory Animals (National Institutes of Health publication 85-23). Male ZO rats from Charles River Laboratories were used for these studies (*n* = 26 rats). All animals were given numbers after randomization based on body weight and before starting treatments and only the numbers were used for identifying the animals during procedures to ensure studies/analyses were performed in a blinded manner. Male Zucker lean (ZL) rats (*n* = 7) were used to compare metabolic parameters. 11-weeks of age, and presence of obesity and pre-diabetes as determined by body weight and fasting glucose levels before the beginning of treatments were the inclusion criteria. Only male ZO rats were used in this study since female ZO rats at this age and fed with normal chow do not develop hyperglycemia, and the goal of the study was to test the effect of NP-6A4 on obesity- and pre-diabetes-induced heart disease with preserved ejection fraction. To evaluate the effect of a 2-weeks treatment with NP-6A4 on cardiac structure and function, 11-week old ZO rats were used. NP-6A4 dissolved in saline (1.8mg/kg/day; 500µl volume) was delivered subcutaneously *via* daily injection and saline was used as control. Rats were maintained on ad libitum food and water and housed singly at the HSTMVH animal housing facility under standard laboratory conditions. Room temperature was maintained at 21–22°C. Light and dark cycles were for 12h, but animals were entrained to have dark cycle (awake time) during the day and light cycle (sleep time) during the night to prevent loss of their sleep time during the experiments. Fasting plasma profile was determined by collecting blood from the saphenous vein as described previously (Luck et al., 2017; Lum-Naihe et al., 2017). Percentage of HbA1c in blood was measured just before euthanasia in animals fasted for 6h using a DCA Vantage Analyzer (Siemens, Malvern, PA). Urine was collected by placing rats in metabolic chambers for 24h according to the protocol approved by the University of Missouri-Columbia IACUC and stored frozen at −80°C until further use. For glucose, enzymatic creatinine (Diazyme, Poway,California,United States), N-acetyl-β- glucosamynidase (β-NAG), urine protein (Beckman Coulter), and Gamma Glutamyl Transferase (GGT) measurements, commercially available assays were used on the AU680 automated clinical chemistry analyzer as reported previously (Nistala et al., 2017). Electrolytes (Na and K) were measured using ion-specific electrodes on the AU680 as well as we have done before (Nistala et al., 2017). Tissues were harvested at the time of euthanasia as described before (Luck et al., 2017), weighed, flash frozen in liquid nitrogen, and stored at −80°C for future use. Wet weights of heart, liver, lung and kidney were determined at the time of euthanasia.

### Echocardiography

Transthoracic echocardiography on saline- or NP-6A4-treated ZO rats was performed under inhaled isoflurane anesthesia (1.5–2.0% maintenance) utilizing a Vevo2100 dedicated rodent ultrasound imaging system (FUJIFILMS, Visualsonics, Toronto) with an MS250S high frequency echo probe at the Small Animal Ultrasound Imaging Center at the Harry S Truman VA Research Center as described previously (Lum-Naihe et al., 2017). Speckle-tracking based strain analysis of B-Mode ultrasound images was performed in the parasternal long- and short-axis views (PLAX and SAX, respectively), as described previously (Lum-Naihe et al., 2017). Images were acquired at the highest frame rates possible (>200 frames per second). PLAX views were used for evaluation of longitudinal strain and strain rate. SAX views that were acquired at the mid-papillary level, were used for evaluation of circumferential and radial strain analyses. Strain analyses were conducted by following the procedure described previously (Lum-Naihe et al., 2017) by using the manufacturer supplied speckle-tracking algorithm (VevoStrain®, VisualSonics). Briefly, at least three of the highest quality B-mode loops were chosen, i.e., those with little gel artifact or obstruction from ribs, as well as those that display the endocardial and epicardial borders throughout the cine loop for each animal. Initially, the endocardial and epicardial borders were traced with the cine loop stopped at end diastole. Cine-loops were replayed to confirm good border tracking over all cardiac cycles and tracking adjustments were made as needed. The final tracked images were then evaluated for strain measurements. Strain measures were averaged over the cardiac cycles yielding curvilinear strain and strain rate data. Global strain values, peak strain and strain rate measurements of NP-6A4 treated rats were compared to those of saline-treated rats. These procedures for data analysis were repeated independently by two qualified individuals for additional confirmation. Blood pressure was measured non-invasively on conscious rats using a CODA volume pressure recording tail-cuff system (Kent Scientific Corporation, Torrington, CT) as described previously (Sharma et al., 2020). All analysis was done using animal numbers to ensure blinded data analysis.

### Histopathology

Tissues from animals (randomly numbered) were fixed in 10% neutral buffered formalin (NBF), embedded into paraffin blocks, sections were cut at 4 µm thickness and used for histopathology as described previously (Luck et al., 2017). To determine interstitial fibrosis, heart sections were stained with Picrosirius Red at Research Animal Diagnostic Laboratory (RADIL), Columbia, MO. The stained sections were scanned using the Aperio CS Slide Scanner by WSI Analytics Lab, Department of Pathology and Anatomical Sciences, University of Missouri, Columbia, MO. Scanned sections were visualized using Aperio ImageScope (Leica Biosystems). Next, 10 interstitial (×20 magnification) images of the most fibrotic regions were selected per animal. Fibrotic area was quantified using the in-built Positive Pixel Count (V9) algorithm (settings were manually determined as follows: hue value = 0; hue width = 0.154; color saturation threshold = 0.04). Positivity (Positive/Total Pixels) was averaged over all regions from a single group to determine mean fibrotic area per group.

To determine cardiomyocyte size and capillary density, heart sections were dewaxed in CitriSolv (Fisher Scientific), rehydrated in an ethanol series and HEPES wash buffer, followed by a heat-mediated antigen retrieval step in sodium citrate buffer. To block non-specific binding sites, sections were incubated with blocking buffer (10% donkey serum, 1% BSA) for 2 h at room temperature, followed by incubation with *Helix pomatia* agglutinin (HPA) conjugated to Alexa Fluor 647 (Life Technologies; 1:400, 2.5 μg/ml) and *Griffonia simplicifolia isolectin* B4 (IB4) conjugated to Alexa Fluor 594 (Life Technologies; 1:200, 5 μg/ml) for 4 h at room temperature. Sections were thoroughly washed and slides were mounted using Fluoroshield with DAPI (Sigma). Imaging was performed using a Leica DMI4000B inverted confocal microscope at 40X and 63X. Cell size and capillary density analysis was done using code written in-house using the EBImage package in R (Pau et al., 2010). Briefly, for measuring cardiomyocyte cell size, HPA stained images were thresholded by intensity to produce binary masks. The masks were used to select closed contours as potential cells. Objects of interest were filtered by morphological parameters (such as area, perimeter, and eccentricity), and subsets of the remaining cells were randomly selected for inclusion in analysis. Following the initial segmentation, artifacts (such as merged cells, split cells, or recognition of non-cell objects) were identified and removed by a user. Area was then computed for approved cells. Cell sizes of a minimum of 65 cells per animal were determined and a minimum of five animals were used per group. A similar protocol was used to extract capillaries for IB4 stained images. Following initial extraction, errors in capillary recognition were manually corrected by a user. All capillaries present in an image were used to calculate the final density. Data are presented as mean ± SEM.

### Immunohistochemistry

Five micrometer heart sections from the saline and NP-6A4 treated ZO rats were deparaffinized in xylene (Fisher Scientific), rehydrated in an ethanol series and HEPES wash buffer, followed by a heat-mediated antigen retrieval step in sodium citrate buffer. Following rehydration, sections were incubated with Wheat germ agglutinin (WGA) conjugated to Alexa Fluor 647 (Life Technologies; 1:200, 5.0 μg/ml). After washing, tissue sections were permeabilized with 0.2% Triton-X (Thermo-Fisher) in PBS and subsequently blocked as described above independently for each AT2R, ACE2, and MAS staining. Antibodies for AT2R (Ab19134), ACE2 (Ab87436), and MAS (Ab66030) were from Abcam Biotech. Sections were thoroughly washed and slides were mounted using Fluoroshield with the nuclear counterstain, DAPI (Sigma). Imaging was performed using a Leica DMI4000B inverted confocal microscope at 20X and 63X. Fluorescence intensity (relative to nuclei) was subsequently quantified using ImageJ software (NIH, Bethesda, MD) using a minimum of 10 regions of interest per section.

### Intracardiac Cytokine Analysis Using Quantibody® Rat Cytokine Array 67 and IPA Analysis

Heart tissues that were frozen at −80°C were homogenized and lysates were prepared for Raybiotech’s Quantibody® Rat Cytokine Array 67 analysis as described previously (Lum-Naihe et al., 2017). Cytokine analysis of the heart tissue lysates was performed by Raybiotech according to their protocol and software analysis. Analysis was performed for 67 non-overlapping cytokines in quadruplicates per animal along with appropriate positive and negative controls. Cytokines that exhibited statistically significant differences (*p* < 0.05, Paired Student’s *t*-Test) between different groups and at least a 1.5-fold change in either direction (increased or decreased) were selected for input into Ingenuity Pathway Analysis (IPA, Qiagen, Germantown, MD) to identify diseases and functions that were affected. Heatmaps were generated using the ggplot2 package for R (Wickham 2016).

### Intracardiac miRNA Expression Analysis

Total RNA was isolated from the frozen (−80°C) heart tissues of saline- or NP-6A4 treated rats using the *mir*Vana™ miRNA isolation kit and GeneChipTM miRNA 4.0 array was used for determining the expression of cardiac miRNA in these isolated total RNA samples as described previously (Belenchia et al., 2018). Briefly, Affymetrix® FlashTag™ Biotin HSR RNA Labeling Kit specifically designed for GeneChipTM miRNA array (ThermoFisher Scientific catalog number 90911) was used for preparing the targets (*n* = 5 animals per group). Hybridization of the target to the GeneChipTM miRNA 4.0 array for 16 h followed by scanning of the fluorescence intensity emitted by the labeled cRNA/cDNA bound to the probe arrays using the Affymetrix GeneChip scanner 3,000 7Gw was performed at the University of Colorado Microarray Core Lab for a fee. Data were collected using Affymetrix Command Console Software at the University of Colorado Microarray Core Lab. Affymetrix Expression Console TM software was used to process the CEL files and normalize data. A two-sample *t*-test was used to compare the two groups for each miRNA and identify the differentially expressed miRNAs that showed statistical significance (*p* < 0.05). The heatmap was generated using the ggplot2 package for R (Wickham 2016).

### RNA Isolation and Quantitative Real Time-PCR

Cardiac expression of *Agtr2* (AT2R), *Agtr1a* (AT1A)*, Agtr1b* (AT1B), miR-7a-1-3p, miR-138-5p, miR-148b-3p, and miR-101b-3p miRNAs were determined using mRNA and miRNA isolated from frozen ZO rat heart tissues (*n* = 5 per group) as described previously (Luck et al., 2017; Lum-Naihe et al., 2017; Belenchia et al., 2018). *Agtr2, At1ra, At1rb, and* 18s PCR reactions were performed in triplicate using TaqMan Fast Universal PCR Master Mix (2X) and TaqMan Assays with primers specific to the genes of interest (Applied Biosystems). miRNA real-time PCR reactions were performed in triplicate using either TaqMan Fast Universal PCR Master Mix (2X) (Applied Biosystems) or miScript II Mix (Qiagen). TaqMan Advanced MicroRNA Assays (Life Technologies) primers were used for miR-7a-1-3p and miR-138-5p. miScript II Assay Primers (Qiagen) were used for miR-148-3p, miR-101b-3p, and miR-190a-3p.

### Statistical Analysis

All data were assessed for homoscedasticity and normality by Shapiro-Wilk test. For the comparison of the two groups, unpaired two-tailed t-tests were performed, with Mann-Whitney correction used in cases of non-parametric data. A *p*-value < 0.05 was deemed significant.

## Results

### NP-6A4 Treatment Attenuated Cardiac Diastolic Dysfunction in ZO Rats Without Changing Their Blood Pressure or Other Metabolic Parameters

Mitral E/e′ ratio is a noninvasive measure of left ventricular filling pressure. Elevated E/e′ ratio is considered as a cardinal sign for heart failure with preserved ejection fraction (HFpEF) (Sharp et al., 2010; Zoppini et al., 2018). In diabetic humans, E/e’ ratio is significantly increased compared to non-diabetic controls indicating diastolic dysfunction (Zoppini et al., 2018). A unit rise in the E/e′ ratio was associated with a 17% increment in risk of a cardiac events as shown in a study of 980 participants in the Anglo-Scandinavian Cardiac Outcomes Trial (ASCOT) (Sharp et al., 2010). In healthy rats, E/e’ is in the range of 18–20. In 13-week old obese and pre-diabetic ZO rats treated with saline, average E/e’ was-31.94 ± 3.24. However, in ZO rats treated with NP-6A4, E/e’ was lowered by an average of 8.5 points and a maximum of 25 points compared to saline-treated ZO rats (Figure 1B; Supplementary Table S2). This observation indicated that NP-6A4 treatment mitigated diastolic dysfunction in ZO rats with untreated obesity and T2DM. The myocardial performance index (MPI) is a Doppler-derived index of global LV function and a negative prognostic marker for congestive heart failure when elevated (Arnlov et al., 2004). NP-6A4 treatment significantly lowered MPI also in ZO rats (Supplementary Table S2) further confirming that NP-6A4 is cardio-protective.

**FIGURE 2:**
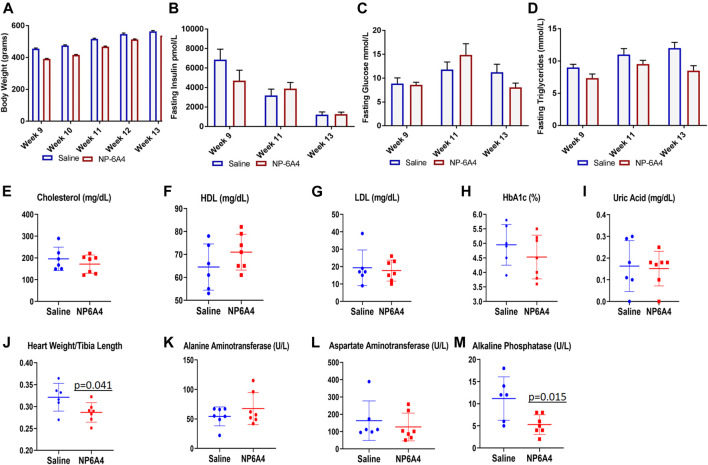
NP-6A4 treatment did not change levels of fasting plasma glucose, insulin, lipids, and aminotransferases, but reduced heart weight and alkaline phosphatase significantly. N = 6 for saline group and seven for NP-6A4 group. Heart weight (grams)/tibia length (cm) and plasma levels of alkaline phosphatase (U/L) at the end of treatment were significantly reduced (*p* values are marked). None of the other parameters showed any significant changes.

NP-6A4 treatment did not change body weight, fasting glucose, insulin, and triglycerides during the course of treatment (Figures 2A–D). Analysis of plasma at the time of euthanasia showed that no significant changes occurred in the levels of cholesterol, high density and low density lipoprotein (HDL and LDL), Hemoglobin A1c Alanine Aminotransferase, Asparatate Aminotransferase and uric acid in response to NP-6A4 treatment (Figures 2E–I). Previous reports show that central infusion with AT2R agonists could reduce blood pressure, however, systemic treatment did not change blood pressure (Sumners et al., 2015; Dai et al., 2016) in different murine models. To verify whether the improvement in cardiac functions of ZO rats resulted from any changes in blood pressure, we tested the effect of four-week NP-6A4 treatment on blood pressure in a separate cohort of ZO rats. Diastolic, systolic and mean arterial pressure were not significantly different between saline- and NP-6A4 treated ZO rats (Supplementary Table S2). This is consistent with other reports that show systemic administration of AT2R agonists are not effective in changing blood pressure. Moreover, this is also consistent with NP-6A4’s effect on blood pressure in a mouse model for aneurism (Sharma et al., 2020). Thus, the NP-6A4-mediated effects on cardiac functions are independent of blood pressure.

### NP-6A4 Treatment Improved Myocardial Global Circumferential Strain and Longitudinal Strain, Reduced Cardiac Fibrosis, Heart Weight and Cardiomyocyte Hypertrophy in ZO Rats

In patients having heart disease with preserved ejection fraction, impairment of left ventricular global longitudinal strain (GLS) is associated with a 5.6-fold increase in mortality (Blomstrand et al., 2015; Krishnasamy et al., 2015). Moreover, impairment of circumferential strain is a negative prognosticator for heart failure in asymptomatic subjects without previous clinical CVD (Choi et al., 2013). Speckle tracking echocardiography analysis (Lum-Naihe et al., 2017) revealed that myocardial longitudinal strain and strain rate, and circumferential strain and strain rate were significantly improved in NP-6A4 treated rats compared to saline treated rats (Figures 1C,D and Supplementary Table S2).

**FIGURE 3:**
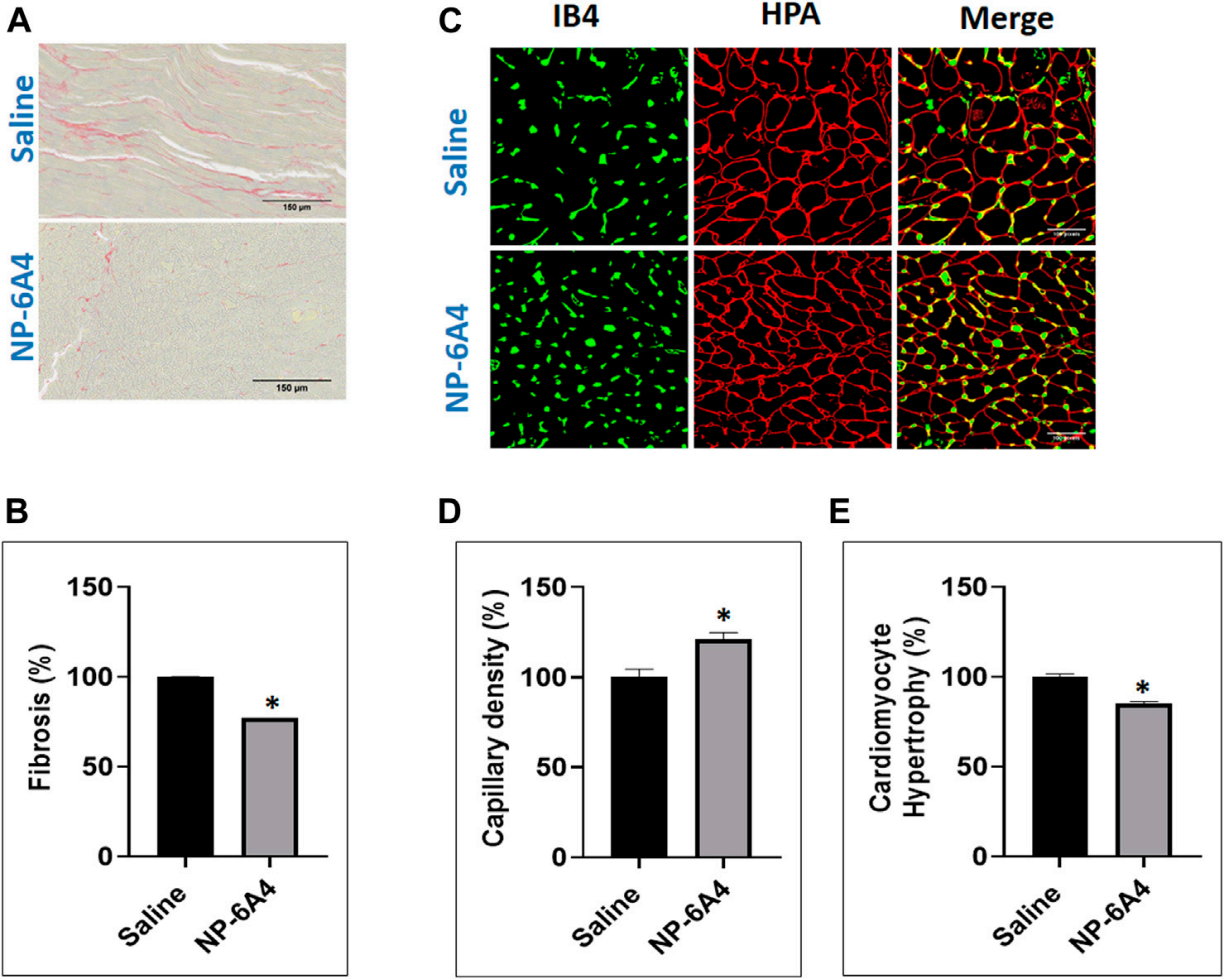
NP-6A4 treatment attenuated cardiac fibrosis, hypertrophy and loss of cardiac microvascular density in ZO rats with untreated diabetes and obesity. **(A)** Representative images of PSR stained heart sections of ZO rats treated with saline or NP-6A4 taken at ×20 magnification (scale bars = 150 µm). **(B)** Graph shows the cumulative data for normalized fibrotic square area (*n* = 6 animals per group; 10 images per animal; 60 images per group). **(C)** Representative images of heart sections co-stained with HPA-Alexa Fluor (red) to visualize cardiomyocyte membrane IB4-Alexa Fluor 594 (green) to visualize capillaries (×40 magnification; scale bars = 100 µm). **(D** and **E)** Graphs show cumulative data for cardiomyocyte size and the number of capillaries per square area. *n* = 6 animals per group; 10 images per animal; and 65 cells per animal from multiple images.

The ZO rats are reported to exhibit cardiac fibrosis starting at the age of 9 weeks (Zhou et al., 2010). The ZO rats used in this study were 13-weeks old at the end of NP-6A4 treatment. Therefore, we tested whether NP-6A4 treatment modulated their cardiac fibrosis. Picrosirius red (PSR) staining (Vogel et al., 2015) was used to visualize collagen content in heart tissue sections from saline- and NP-6A4 treated ZO rats. There was significant reduction in PSR staining in NP-6A4-treated ZO rat hearts (Figures 3A,B). This suggests that NP-6A4 reduces excessive collagen and thereby attenuates cardiac fibrosis. The wet heart weight adjusted to tibia length was significantly reduced in response to NP-6A4 treatment in ZO rats (Figure 2J) indicating suppression of cardiac hypertrophy. Cardiomyocyte hypertrophy is often seen in obese and diabetic patients (Oktay et al., 2000) and reported in ZO rats (Martinelli et al., 2020). To test if NP-6A4 modulated cardiomyocyte hypertrophy in obese and pre-diabetic ZO rats, we performed immunofluorescent cell membrane staining using *Helix pomatia* agglutinin (HPA) conjugated to Alexa Fluor 647 to evaluate cardiomyocyte size (Lum-Naihe et al., 2017). As shown in Figures 3C,D, NP-6A4 treated hearts had a significant reduction in cardiomyocyte size indicating that the short-term NP-6A4 treatment could reduce cardiomyocyte hypertrophy.

### NP-6A4 Treatment Increased Cardiac Capillary Density in ZO Rats

In patients with chronic heart failure clinical research has shown that there is a ∼20% reduction in their skeletal muscle capillary density and that was inversely related to their maximum oxygen consumption (Duscha et al., 1999). It is also shown that chronic diabetes induces loss of capillary density (capillary rarefaction) and that diabetic human myocardial explants exhibit significant capillary rarefaction and pericyte loss compared to nondiabetic explants (Hinkel et al., 2017). Therefore, we tested whether NP-6A4 treatment modulated cardiac capillary density in obese and pre-diabetic ZO rats. Co-staining of the HPA-stained sections with the vascular stain Isolectin B4 (IB4) to determine capillary presence showed that NP-6A4 treatment increased IB4 staining by 20.8% suggesting that NP-6A4 increased cardiac capillary density in ZO rats (Figures 3C,E). Given the significance of 20% capillary rarefaction in muscle pathology of diabetic patients, NP-6A4-mediated increase of 20.8% cardiac capillary density in ZO rats with untreated obesity and pre-diabetes is likely a significant contributor to their improved cardiac function in response to NP-6A4-treatment. Moreover, increase in capillary density is consistent with NP-6A4-induced improvement of human coronary endothelial cell function (Toedebusch et al., 2018).

### NP-6A4 Activated a Feed-Forward Loop That Increased Cardiac AT2R Expression

**FIGURE 4:**
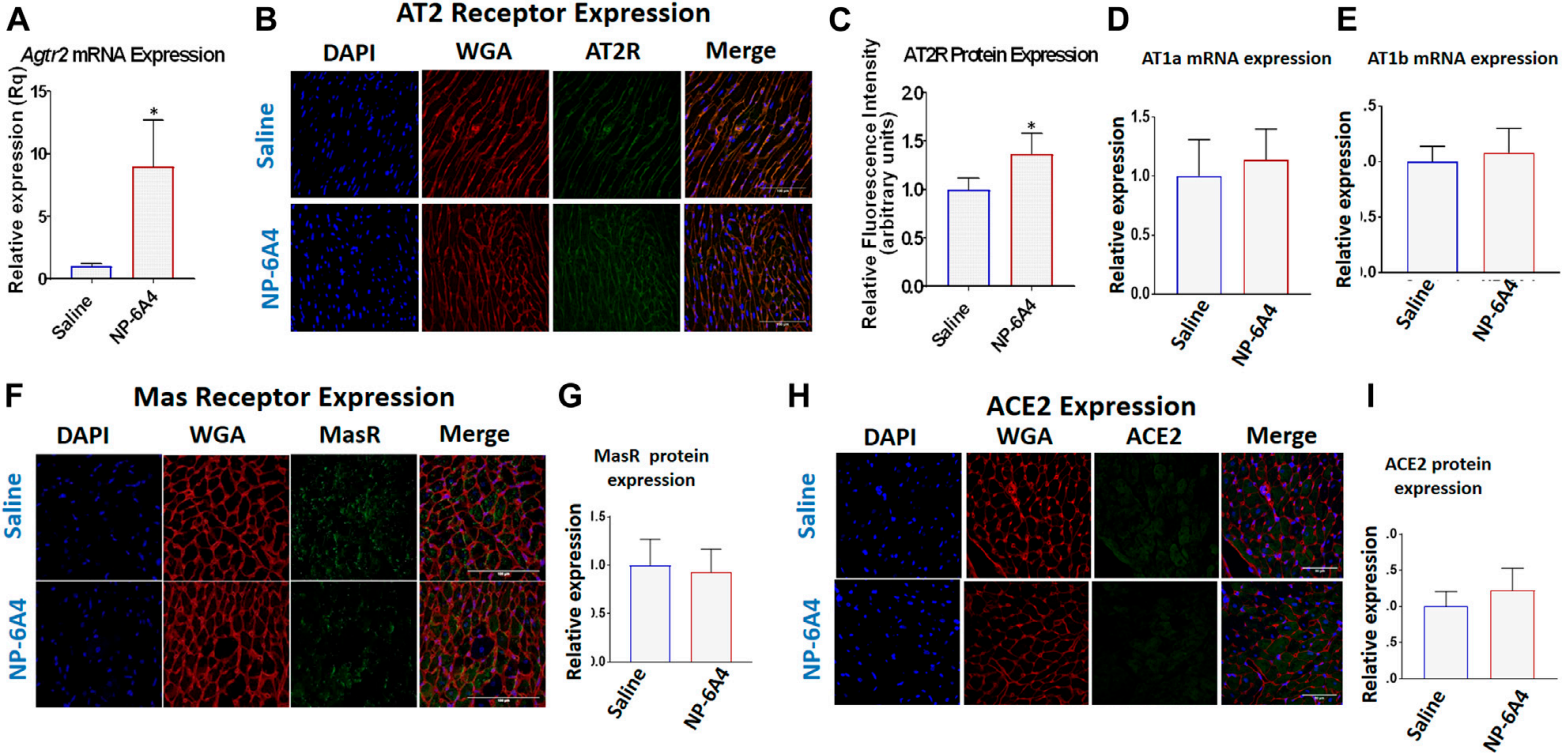
NP-6A4 treatment increased expression of cardiac AT2R, but not AT1 receptor subtypes, Mas receptor or ACE2. **(A)** Expression of cardiac *Agtr2* (AT2R) mRNA as determined by qRT-PCR in Saline-and NP-6A4 treated rats. *n* = 6 per group. **(B)**. Representative images of heart sections from immunohistochemistry analysis using anti-AT2R antibody as the probe (green), nuclei visualized by DAPI staining (blue) and cell membranes visualized by staining with WGA-Alexa Fluor 647 (red) (×63 magnification, scale bars = 50 µm). **(C)** Graphs show cumulative data for normalized anti-AT2R antibody staining. **(D** and **E)** Expression of cardiac AT1 receptor subtypes AT1a and AT1b mRNA expression as determined by qRT-PCR. **(F** and **H)** Representative images of heart sections from immunohistochemistry analysis using anti-MasR antibody or anti-ACE2 antibody as the probe (green), nuclei visualized by DAPI staining (blue) and cell membranes visualized by staining with WGA-Alexa Fluor 647 (red). **(G** and **I)** Graphs show cumulative data for normalized anti-MasR antibody and anti-ACE2 antibody staining.

AT2R agonists CGP42112A and C21 do not increase AT2R expression in the heart and are not used to treat heart disease (Wan et al., 2004; Chow et al., 2016). We reported that NP-6A4 increased AT2R expression in human cardiovascular cells (Toedebusch et al., 2018). Consistent with this, NP-6A4-treatment increased *Agtr2* mRNA expression by at least 5-fold in ZO rat hearts as assessed by quantitative RT-PCR (Figure 4A). Immunohistochemistry analysis using anti-AT2R-antibody also showed an increase in AT2R protein expression in NP-6A4-treated ZO rat hearts (Figures 4B,C). However, protein expression levels of other RAAS components (Gul et al., 2012) such as AT1 receptor subtypes AT1A (Figure 4D) and AT1B (Figure 4E), or anti-inflammatory RAAS components such as MAS receptor (Figures 4F,G) and ACE2 (Figures 4H,I) did not change in response to NP-6A4 treatment in ZO rat hearts.

### NP-6A4 Induced Suppression of a Large Array of Inflammatory and Pro-fibrotic Cytokines and Chemokines in ZO Rat Heart

**FIGURE 5:**
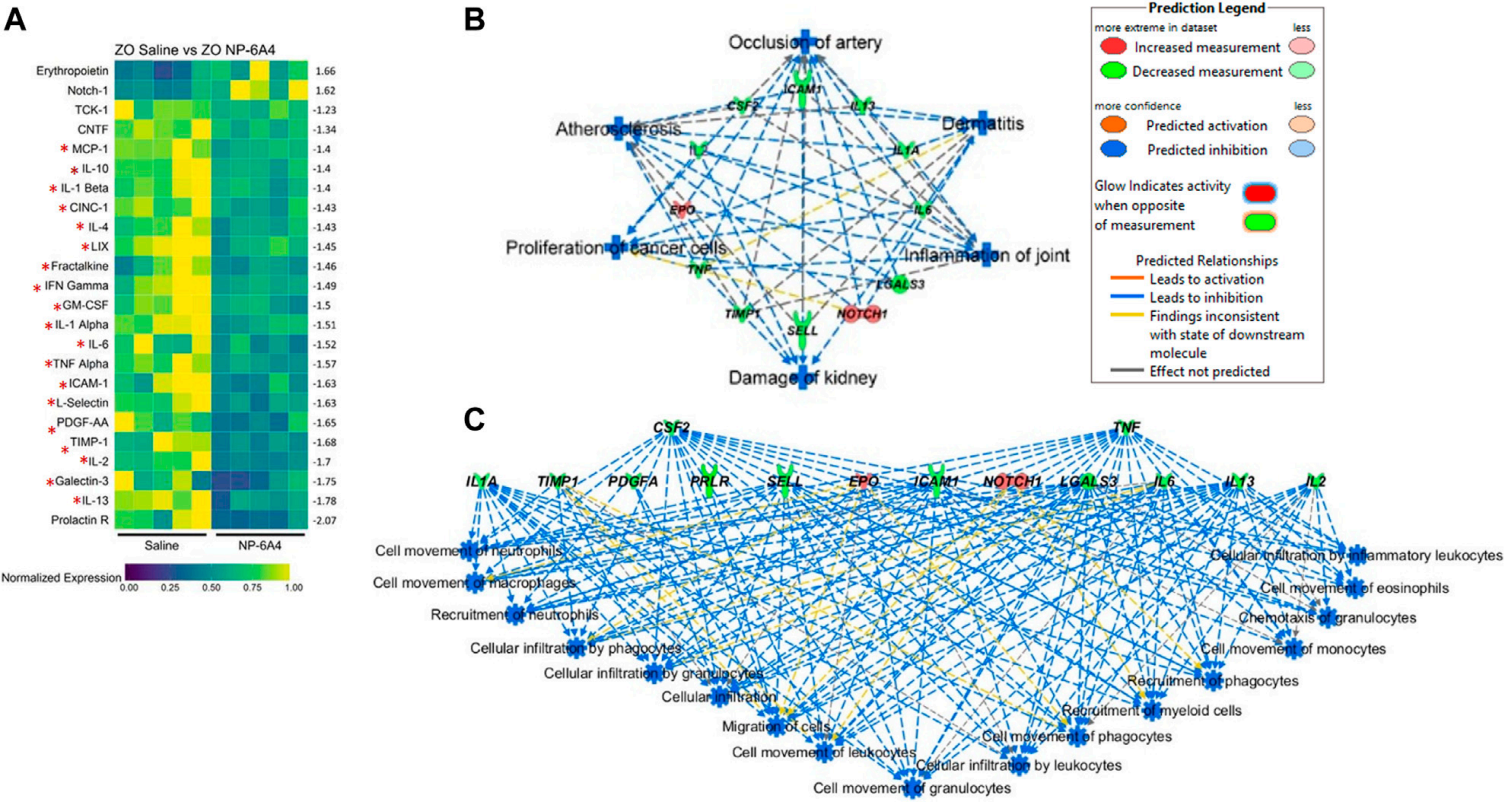
NP-6A4 suppressed a large array of inflammatory and fibrotic cytokines implicated in both cardiac pathologies and cytokine storm and simultaneously increased expression of cardio-protective erythropoietin (EPO) and Notch1 that promote myocardial repair in the heart tissues of obese and pre-diabetic ZO rat. **(A)** Heat map shows a graphic representation of relative expression of 24 differentially expressed intracardiac cytokines significantly different between saline and NP-6A4 treated ZO rat hearts. Individual cardiac samples are arranged along the *x*-axis and cytokines along the *y*-axis. 19 cardiac molecules implicated in cardiac pathologies (Table 1) and suppressed by NP-6A4 are marked by red star. Fifteen cytokines that exhibited 1.5-fold differential expression in either direction were further selected for IPA analysis (see Supplementary Table S3). *n* = 5 animals per group. Yellow indicates the highest expression for each cytokine (1.0), while dark blue indicates lower expression relative to the sample with the highest expression. Expression was normalized for each cytokine across all animals (across each row). Statistical significance was determined using Student’s t-test. *p* < 0.05 for all proteins. **(B)** Top scoring IPA-predicted diseases and functions networks resulting from the comparison of cytokine proteins (Supplementary Table S3) of saline and NP-6A4 treated ZO rat hearts. Six diseases and functions were predicted to be suppressed (blue in color) by NP-6A4 treatment based on this cytokine profile. Additional details for each individual network in this group are provided in Supplementary Figure S2. **(C)** Immune cell specific functions that are predicted to be suppressed in NP-6A4 treated ZO rat hearts. Green cytokines indicate downregulation in NP-6A4-treated compared with saline-treated ZO rat hearts. The blue connecting lines show a known relationship between the cytokine and the cellular process/function. This figure was generated using IPA’s built in feature for “Diseases and Functions”, and then selecting “immune system related” processes.

To gain a better understanding of the mechanisms underlying the cardioprotective effects of NP-6A4-AT2R signaling we analyzed the intracardiac cytokine profile using the Quantibody® Rat Cytokine Array 67 analysis of RayBiotech. Inc. (performed by RayBiotech). Twenty four cytokines were significantly differentially expressed in NP-6A4-treated compared to saline-treated ZO rat hearts (Figure 5A). Among them were 19 pro-inflammatory and pro-fibrotic cytokines indicating that NP-6A4 activates a unique anti-inflammatory signaling that coordinately suppresses several inflammatory cytokines that contribute to the complex cardiac pathology caused by obesity and pre-diabetes. Table 1 shows the roles of interleukins IL-1α and β, IL-2, IL-4, IL-6, and IL-13, Fractalkine, Interferon-γ (IFN-γ), Tumor necrosis factor-α (TNF-α), Monocyte chemotactic protein-1 (MCP-1), Galactin-3, Granulocyte-monocyte-colony stimulating factor (GM-CSF), Cytokine-induced neutrophil chemoattractant 1 (CINC-1, also known as CXCL1), and Intracellular Adhesion Molecule 1(ICAM-1), in cardiac pathology and are established from clinical and pre-clinical studies. These cytokines are specifically implicated in fibrosis, hypertrophy, cardiomyopathy, coronary heart disease, or heart failure in humans and animal models. LIX, also known as CXCL5, is increased in autoimmune diseases. As shown in Figure 5A, all of these inflammatory cytokines are significantly suppressed by NP-6A4 treatment in ZO rat heart. Interestingly, these same cytokines are also implicated in cytokine storm induced by infectious diseases including COVID-19 and they are drug targets for mitigating cytokine storm-induced tissue damage (Table 1). Additionally, interleukin-10 (IL-10) secreted by cardiac macrophages has a critical role in cardiac fibrosis specifically in heart failure with preserved ejection fraction (HFpEF) (Hulsmans et al., 2018). NP-6A4 treatment suppressed cardiac IL-10 also in ZO rats (Figure 5A). L-selectin is a cell adhesion molecule that shows increased expression in inflamed vascular tissues and is implicated in leukocyte infiltration in cardiovascular diseases (Weil and Neelamegham 2019). NP-6A4 treatment suppressed cardiac L-selectin levels also (Figure 5A). Platelet-derived growth factor A-chain (PDGF AA) that is increased during vascular hypertrophy and COVID-19 (Zhao et al., 2011; Petrey et al., 2021) and Tissue inhibitor of metalloproteinases (TIMP1) that promotes myocardial fibrosis (Takawale et al., 2017) are also suppressed by NP-6A4 (Figure 5). Other molecules suppressed by NP-6A4 include TCK1 and Prolactin receptor, however their potential roles in cardiac functions in conditions of obesity and pre-diabetes are yet to be elucidated.

**TABLE 1:** Established roles of cytokines and chemokines suppressed by NP-6A4 in ZO rat heart in different pathologies.

Cardiac cytokines/chemokines suppressed by NP-6A4	Increased expression levels in							Inhibitors used in clinic for heart disease/COVID-19	References
	Myocardial hypertrophy	Cardiac fibrosis/leukocyte infiltration	Cardio-myopathy/myocarditis	Myocardial infarction/Heart failure	Obesity/Diabetes	Auto-inflammatory diseases	Cytokine storms/COVID-19		
IL-1α and β	Yes	Yes	Yes	Yes	Yes	Yes	Yes	Anakrina	Abbate et al. (2020); Van Tassell et al. (2018); Conti et al. (2020); Peiró et al. (2017)
IL-2	-	-	Yes	-	Yes	Yes	Yes	-	Thavendiranathan et al. (2011); Mangalmurti and Hunter. (2020); Frati et al. (2017)
IL-4	-	Yes	Yes	-	Yes	-	Yes	-	Kanellakis et al. (2012); Diny et al. (2017); Schmidt et al. (2015); Fara et al. (2020)
IL-6	Yes	Yes	Yes	Yes	Yes	Yes	Yes	Tocilizumab	Bacchiega et al. (2017); Mangalmurti and Hunter. (2020); Zhang et al. (2016); Chen et al. (2020)
								Sarilumab	
								Siltuximab	
IL-13	-	Yes	-	-	-	-	Yes	-	Costela-Ruiz et al. (2020); Liu et al. (2015)
Fractalkine	-	Yes	Yes	Yes	Yes	Yes	Yes	KAND567*	Costela-Ruiz (et al. (2020); Xuan et al. (2011); Kikuchi et al. (2004)
IFN-γ	-	-	Yes	Yes	Yes	-	Yes	-	Costela-Ruiz et al. (2020); Torzewski et al. (2012); Reifenberg et al. 2007
TNF-α	Yes	Yes	Yes	Yes	Yes	Yes	Yes	Remicade, Enbrel, Humira, Cimzia, Simponi	Costela-Ruiz et al. (2020); Schumacher and Naga Prasad (2018)
MCP-1	Yes	Yes	Yes	Yes	Yes	Yes	Yes	-	Costela-Ruiz et al. (2020); Rolski and Błyszczuk (2020); Niu and Kolattukudy. (2009)
Galactin-3	-	Yes	Yes	Yes	Yes	Yes	Yes	-	Costela-Ruiz et al. (2020): Souza et al. (2017); Vora et al. (2019); Caniglia et al. (2020)
GM-CSF	Yes	Yes	Yes	Yes	Yes	Yes	Yes	Lenzilumab	Costela-Ruiz et al. (2020): Anzai et al. (2017)
CINC1 (CXCL1)	Yes	Yes	Yes	Yes	Yes	Yes	Yes	-	Costela-Ruiz et al. (2020): Zagorski et al. (2007); Wang et al. (2018)
ICAM1	Yes	Yes	Yes	Yes	Yes	Yes	Yes	Xiidra (lifitegrast)	Costela-Ruiz et al. (2020): Salvador et al. (2016); Luc et al. (2003)
LIX (CXCL-5)	-	-	-	-	-	Yes	Yes	-	Costela-Ruiz et al. (2020): Koenig et al. (2020)
IL-10	-	Yes	-	Yes	Yes	-	Yes	-	Hulsmans et al. (2018)
L-Selectin	-	Yes	-	-		-	?		Weil and Neelamegham. (2019)
PDGF-AA	-	Yes	Yes	Yes	Yes	-	Yes	-	Zhao et al. (2011); Petrey et al. (2021)
TIMP-1	-	Yes	-	-	-	-	-	-	Takawale et al. (2017)

### NP-6A4 Treatment Increased the Expression of Cardioprotective Erythropoietin (EPO) and Notch1 in Addition to the Reparative AT2R

Cardiac levels of two molecules implicated in cardiac repair, Erythropoietin (EPO) (Santhanam et al., 2010; Zafiriou et al., 2014)) and Notch1 (Yu and Song 2014; Zhu et al., 2020; Li et al., 2021) were significantly increased by NP-6A4 in ZO rat heart (Figure 5A). Thus, NP-6A4 increased the expression of at least three cardioprotective molecules in the ZO rat heart, namely, AT2R, EPO and Notch1. Increase in the expression of these protective molecules combined with suppression of a large array of pro-inflammatory and pro-fibrotic molecules by NP-6A4 indicate that NP-6A4 treatment induces an unprecedented cardioprotective cytokine network in the heart tissues of ZO rats even in the presence of their untreated obesity and pre-diabetes.

### Ingenuity Pathway Analysis Predicts NP-6A4-AT2R-Induced Cardiac Cytokine Network is Cardiovascular and Renal Protective

To gain a better insight into how NP-6A4 induced cytokine network modulates cardiovascular pathology, we selected cytokines that had at least 1.5-fold change in either direction (Supplementary Table S3) and used them as input for Qiagen’s Ingenuity Pathway Analysis (IPA) software. According to IPA prediction, NP-6A4-induced cytokine network could significantly suppress the following diseases and functions: 1) occlusion of artery, 2) atherosclerosis, 3) damage to kidney, 4) inflammation of joint, 5) proliferation of cancer cells and 6) dermatitis. (*p* > 0.001; Figure 5B). Details on activation Z score and number of molecules in each pathway are shown in Supplementary Figure S1). Additionally, IPA predicted significant suppression of inflammatory response by NP-6A4 in ZO rat heart (Figure 5C). These results of IPA analysis further suggest that there is activation of a unique cardioprotective and anti-inflammatory cytokine signature by NP-6A4 in ZO rat heart in conditions of untreated obesity, hyperlipidemia, and T2DM. This is a key mechanism for NP-6A4’s ability to attenuate myocardial functional and structural damage resulting from obesity and pre-diabetes.

### Other Systemic Effects of NP-6A4: Suppression of Urinary β-NAG, Lung Weight Adjusted to Tibia Length, and Plasma Alkaline Phosphatase

While NP-6A4 treatment did not change the 24-h urine output significantly (Table 2), it reduced the total amount of N-acetyl-β- glucosamynidase (β- NAG) by 66% in 24-h urine sample of ZO rats (Table 2). Urinary β- NAG is a marker for tubulointerstitial injury in diabetes (Kim et al., 2016). Nephroprotective treatments such as with acetaminophen is shown to reduce β- NAG in the urine of ZO rats by 41% (89). Therefore, our observation that NP-6A4 reduced urinary β- NAG in a 24-h urine sample indicates that NP-6A4 treatment is nephroprotective in ZO rats. NP-6A4 also induced a modest, but significant reduction in total enzymatic creatinine in the 24-h urine sample (Table 2). Other urine metabolites (sodium, potassium, glucose, Gamma-Glutamyl-Transferase, and protein) did not change significantly (Table 2). Additionally, as shown in Figure 2; Table 2, heart, kidney and liver wet weights adjusted to tibia length were not significantly different between ZO rats treated with saline or NP-6A4. However, wet weight of lung was reduced by 15% (*p* = 0.023) in response to NP-6A4 in ZO rats (Table 2). This observation indicated that NP-6A4 treatment modulated lung weight in an animal model that exhibits diastolic dysfunction with preserved ejection fraction, obesity and pre-diabetes. Lung congestion is associated with chronic heart failure (Melenovsky et al., 2015). However, additional studies are needed to fully evaluate the role of NP-6A4 on lung complications induced by obesity and chronic inflammation. ZO rats are reported to have elevated plasma alkaline phosphatase, a marker of liver and bone injury (Gary-Bobo et al., 2007). NP-6A4 treatment significantly reduced fasting plasma alkaline phosphatase (Figure 2M) indicating that NP-6A4 treatment may be protective for liver and bone tissues.

**TABLE 2:** Effects of NP-6A4 treatment on urine parameters and wet weights of other tissues than heart in Zucker obese rats with untreated diabetes, obesity, and metabolic syndrome.

Treatment	Saline	NP-6A4	*p* value
**Metabolites (total amount in 24 h urine)**			
24-h urine (ml)	36 ± 6	41 ± 6	0.45
Na^+^ (mEq)	3.4 ± 0.4	3.0 ± 0.3	0.62
K^+^ (mEq)	7.8 ± 1.07	5.25 ± 0.46	0.08
Glucose (mg)	1,433 ± 486	1,343 ± 372	0.53
Gamma-glutamyl transferase (GGT) (U)	12.11 ± 3.8	12.09 ± 2.2	0.92
N-acetyl-β-glucosamynidase (β-NAG) (mU)	879.2 ± 128	385.3 ± 99	0.017
Protein (mg)	412 ± 130	191 ± 48	0.07
Enzymatic creatinine	219.5 ± 24	86.6 ± 8.5	0.0006
**Wet tissue weights (gram) adjusted to tibia length (cm)**			
Lung	0.337 ± 0.06	0.286 ± 0.06	0.02
Liver	7.11 ± 0.58	6.08 ± 0.55	0.16
Kidney	1.06 ± 0.014	0.88 ± 0.15	0.056

### NP-6A4 Increased miRNAs That Inhibits Cardiac Fibrosis and Hypertrophy and Suppress Inflammatory Cytokines

**FIGURE 6:**
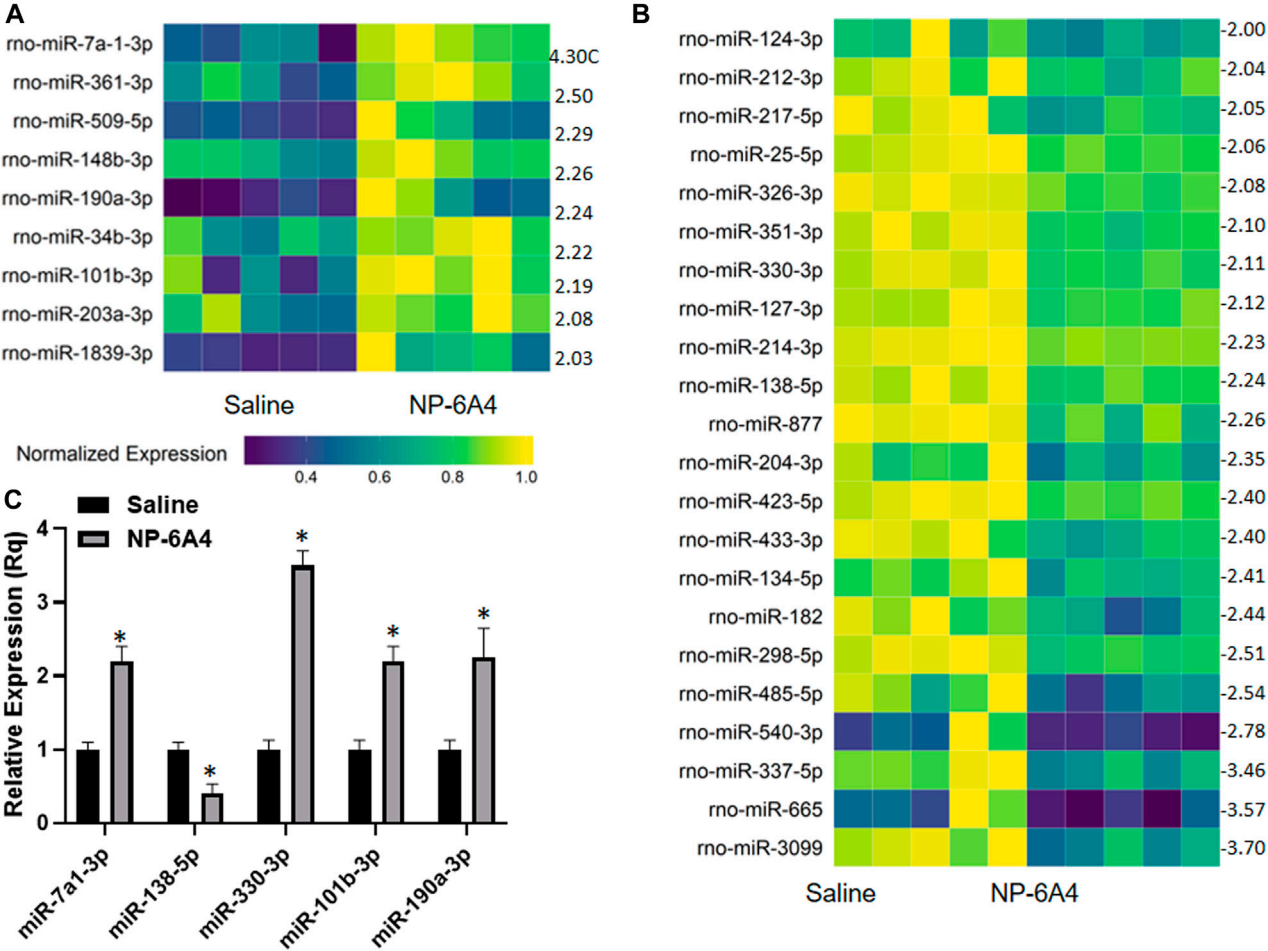
NP-6A4 modulates a cardiac microRNAs that can potentially regulate the anti-inflammatory protein network in ZO rat heart. **(A** and **B)** Heat maps show graphic representation of relative expression of intracardiac microRNAs that exhibited at least a two-fold increase **(A)** or two-fold decrease **(B)** in NP-6A4-vs. saline-treated ZO rat hearts. *n* = 5 animals per group. Yellow indicates the highest expression for each cytokine (1.0), while dark blue indicates lower expression relative to the sample with the highest expression. Expression was normalized for each microRNA across all animals (across each row). Statistical significance was determined using Student’s *t*-test. *p* < 0.05 for all differentially expressed miRNAs. Supplementary Tables S4A,B list all miRNAs that exhibited at least 1.5-fold differential expression. **(C)** Graph shows the results of quantitative RT-PCR analysis of some of the differentially expressed miRNAs in NP-6A4 vs. saline treated ZO rat hearts previously identified by microarray analysis. *n* = 6 animals per group and qRT-PCR was performed in triplicates. *p < 0.05 for saline-treated vs. NP-6A4 treated as determined by two-tailed paired *t*-test.

**FIGURE 7:**
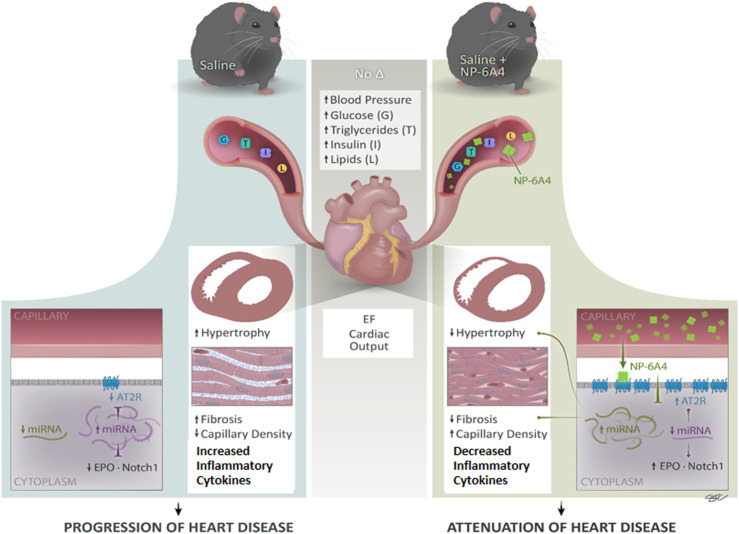
NP-6A4 mitigates heart disease *via* a new multi-prong signaling that coordinates expression of cardioprotective protein and miRNA in conditions of untreated obesity and pre-diabetes. Cartoon diagram summarizes the key events underlying cardioprotection induced by NP-6A4-AT2R signaling in untreated diabetes and obesity. NP-6A4 treatment attenuated cardiac dysfunction as evidenced by reduced E/e’, improved GLS and GCS. NP-6A4 suppressed myocardial structural damage as evidenced by reduction in fibrosis, cardiomyocyte hypertrophy, and loss of microvascular density. These protective effects were mediated by a coordinated increase in the expression of cardio-reparative AT2R, EPO and Notch1 (marked in the diagram) and suppression of nineteen pro-inflammatory and/or pro-fibrotic cytokines. Expression of multiple miRNAs that can potentially target AT2R, EPO and Notch1 (shown in purple) are suppressed whereas miRNAs implicated in attenuating heart failure, hypertrophy and fibrosis and suppressing inflammatory cytokines (shown in light green) are increased by NP-6A4-AT2R signaling. Since all these cardioprotective effects are induced in conditions of untreated obesity, and without modulating blood pressure, NP-6A4 can be an exciting synergistic drug for anti-hyperglycemic, anti-lipidemic and anti-hypertensive drugs to mitigate severe cardiac inflammation and heart disease.

MicroRNAs have emerged as powerful diagnostic markers for heart disease and master regulators of gene expression. Therefore, we analyzed NP-6A4-induced changes in miRNA expression pattern in ZO rat hearts. Analysis of miRNA expression patterns using the Affymetrix miRNA GeneChip Version 4.0 identified 95 miRNAs that were significantly differentially expressed by at least 1.5-fold (Supplementary Tables S4A,B), of which 31 miRNAs showed a 2-fold differential expression (Figures 6A,B). Quantitative RT-PCR analysis was used to spot check the expression patterns of some of these miRNAs as an alternative method to verify the data from microarray. All miRNAs tested by qRT-PCR confirmed the differential expression pattern seen in microarray analysis (Figure 6C). Next we checked whether any of the miRNAs that showed increased expression were reported to have cardioprotective effects according to literature. We found that miR-101b-3p abrogates cardiomyocyte hypertrophy and fibrosis (Pan et al., 2012; Lee et al., 2017) and miR-148b-3p, is downregulated in mitral regurgitation and heart failure (Chen et al., 2016; Figure 6A; Table 3). Literature also showed that miR-101b-3p suppresses Stc1 (Stanniocalcin 1) implicated in macrophage differentiation, preferentially M1 polarization, and contributes to the increase in expression of multiple inflammatory cytokines (Table 3; De Martino et al., 2009; Leung and Wong, 2021) Similarly, miR-190a-3p is shown to target and suppress Tiam1 that activates *il17* promoter and increase inflammatory cytokines (Table 3; Kurdi et al., 2016. Liu et al., 2019). It is conceivable that up-regulation of miRNAs that suppress different positive regulators of inflammatory cytokines can be one potential mechanism underlying this coordinated suppression of a large array of inflammatory cytokines by NP-6A4 (Figure 7). However, additional studies are needed to test this idea.

**TABLE 3:** Differentially expressed cardiac miRNAs of ZO rats in response to NP-6A4 treatment may contribute to mitigating cardiac damage, suppressing inflammatory cytokines and regulating expression of AT2R, EPO and Notch1.

miRNA	Fold change in NP-6A4 treated rat hears as per microarray analysis	Confirmed and/or predicted function
miR-148-b3p	**2.2**	Associated with decrease in mitral regurgitation Chen et al. (2016)
miR-101b-3p	**2.18**	Associated with decrease in cardiac fibrosis and hypertrophy Pan et al. (2012); Lee et al. (2017). Targets and suppresses Stc1 implicated in M1 macrophage polarization and increasing expression of inflammatory cytokines De Martino et al. (2009); Leung and Wong (2021)
miR-190a-3p	**2.23**	Shown to suppress Tiam1 that increases expression of inflammatory cytokines Kurdi et al. (2016); Liu et al. (2019)
miR-182	**−2.43**	Decreased expression → increased expression of EPO, Notch1, and EphA5^a^
miR-138-5p	**−2.23**	Decreased expression → increased expression of AT2R, EPO, and Notch1^a^
miR-330-3p	**−2.1**	Decreased expression → increased expression of AT2R and Notch1^a^
miR-337-5p	**−3.45**	Decreased expression → increased expression of EphA5^a^

a Denotes predicted effects of these miRNA changes on the expression of cardiac proteins based on the in silico analysis using miRDB software.

## Discussion

Results presented here show for the first time that NP-6A4, a peptide agonist of AT2R with an FDA designation for pediatric cardiomyopathy, could suppress an inflammatory cytokine network comprised of 21 proteins in the heart tissues of ZO rats with untreated obesity and T2DM, and ameliorate cardiac structural and functional damage. Previous studies have shown that the presence of either a high E/E′ ratio or an impaired myocardial global longitudinal strain (GLS) provides a useful negative predictive value of cardiovascular events in patients with type 2 diabetes (Liu et al., 2016).

We show that in young obese and pre-diabetic ZO rats that exhibit cardiac dysfunction with preserved ejection fraction, NP-6A4 treatment attenuated early myocardial deformation [as evidenced by improvement of myocardial GLS and circumferential strain (GCS)], and diastolic dysfunction (as evidenced by reduction in E/e’ and MPI). NP-6A4 also suppressed cardiac fibrosis and cardiomyocyte hypertrophy, and increased cardiac capillary density in ZO rats. The mode of action of NP-6A4 involves activating a positive feedback loop that increases expression of its receptor, the cardio-reparative AT2R. However, unlike AT1 receptor blockers (Igase et al., 2008), and other AT2R agonists (Sumners et al., 2015; Dai et al., 2016), NP-6A4 did not increase cardiac levels of ACE2 protein indicating that NP-6A4-AT2R-signaling is unique. Mas receptor protein levels in the heart were also unaffected by NP-6A4.

NP-6A4 treatment did not induce any significant changes in bodyweight, fasting plasma glucose, insulin, triglycerides, and cholesterol, indicating that cardioprotective effects of NP-6A4 did not stem from modulating these parameters. Although central activation of AT2R is involved in reducing blood pressure, systemically applied AT2R agonists do not change blood pressure and AT2R agonists are not used as anti-hypertensive drugs (Sumners et al., 2015; Dai et al., 2016). Consistent with this, we also did not see any significant changes in blood pressure in ZO rats in response to this sub-cutaneous NP-6A4 treatment (Supplementary Table S2). Thus, cardioprotective effects of NP-6A4 do not arise from significant blood pressure reduction. These observations suggest that NP-6A4 can be cardioprotective in the continuous presence of a toxic metabolic profile indicated by hyperglycemia, hyperinsulinemia, and hyperlipidemia.

Capillary loss or rarefaction is a dangerous cardiac pathology observed in human diabetic hearts and skeletal muscle biopsies of patients with chronic heart failure (Duscha et al., 1999; Hinkel et al., 2017). We have reported previously that NP-6A4 increased endothelial function as evidenced by increased expression of endothelial nitric oxide synthase and generation of nitric oxide in human coronary artery endothelial cells (Toedebusch et al., 2018). Therefore, the observed increase in cardiac capillary density in ZO rats could be the result of improved endothelial function induced by NP-6A4-AT2R signaling in ZO rat heart. Such improvement in capillary density is key to improved cardiac function.

Our data show that NP-6A4-AT2R signaling suppresses 19 cardiac cytokines and chemokines involved in pathologic remodeling of the heart (Figures 4A–C). Among them are the IL-1 ligands, IL-1α and β, that act through IL-1 receptor, and implicated in NRLP3 inflammasome-induced cardiac inflammation, diabetic cardiomyopathy, septic shock, myocardial infarction, heart failure and atherosclerosis (Peiró et al., 2017; Abbate et al., 2020). IL-1 receptor blockers or monoclonal antibodies against IL-1β are currently in clinical trials to mitigate cardiac inflammation. Moreover, Anakrina, an IL-1 receptor blocker, is in clinical trials to mitigate cardiac and pulmonary inflammation and death (Cauchois et al., 2020; Chen et al., 2020). IL-2 implicated in myocarditis, and IL-4, IL-6, and IL-13 implicated in fibrosis and cardiomyopathy are the other inflammatory interleukins suppressed by NP-6A4 (Table 1). Fractalkine and Galactin-3 are two other inflammatory cytokines that are implicated in cardiomyopathy and heart failure. Other prominent inflammatory cytokines suppressed by NP-6A4 are IFNγ, TNF-α, MCP-1, GM-CSF, CINC-1 (CXCL-1), ICAM-1, LIX (CXCL1), and PDGF-AA. These data suggest that NP-6A4-AT2R signaling activates a unique regulation of the large array of pro-inflammatory cytokines implicated in pathologic remodeling of cardiovascular tissues in response to metabolic and infectious diseases.

At the same time NP-6A4-AT2R signaling increases expression of Notch1 and EPO that promote cardiac repair. Notch1 signaling is activated in response to myocardial injury and is involved in suppression of cardiomyocyte apoptosis, cardiac repair and regeneration (Li et al., 2010; Yu and Song, 2014; Zhu et al., 2020). Erythropoietin (EPO) increases myocardial performance and induces cardiac repair after myocardial infarction (Santhanam et al., 2010; Zafiriou et al., 2014). Thus, NP-6A4-AT2R signaling induces a coordinated anti-inflammatory cytokine-network that mitigates cardiac pathologic remodeling in ZO rats with untreated obesity and pre-diabetes.

To gain insight into the relationship between the NP-6A4-induced increase in cardioprotective AT2R, EPO and Notch1 and the changes in intracardiac miRNAs, we performed an *in silico* analysis. Interestingly, analysis of 3′untranslated regions of mRNAs coding for human AT2R, EPO, and Notch1 using RegRNA 2.0 software (Chang et al., 2013) showed that multiple miRNAs that are suppressed by NP-6A4 treatment (Figure 6B) had binding sites on the these mRNAs (Table 3; Supplementary Table S5). In contrast, none of the miRNAs that showed increased expression over two-fold in NP-6A4-treated hearts (Figure 6A) had binding sites in the 3′-untranslated regions of these mRNAs. This observation suggested that NP-6A4 mediated suppression of some microRNAs can be one of the underlying mechanisms for the NP-6A4-induced increases in the expression of AT2R, EPO and Notch1 in ZO rat heart. Additional studies are warranted to test this idea.

At the age of 9 weeks, ZO rat heart exhibits diastolic dysfunction, cardiac fibrosis, abnormal cardiomyocyte histoarchitecture, and increased levels of 3-nitrotyrosine, and NADPH oxidase-dependent superoxide compared to Zucker lean rat heart (Zhou et al., 2010). Moreover, another study showed that at the age of 12-weeks, ZO rat heart exhibits increased levels of lipid-aldehyde 4-hydroxynonenal (4-HNE), platelet endothelial cell adhesion molecule-1 (PECAM-1) and vascular cell adhesion molecule-1 (VCAM-1) and TNF-α compared to lean Zucker rats indicating the highly inflammatory status of the ZO rat heart (Martinelli et al., 2020). The ZO rats in this study were 11-week old, an age when cardiac inflammation and damage caused by untreated obesity and pre-diabetes was progressing. NP-6A4-AT2R signaling seems to have mitigated this cardiac inflammation by activating a unique anti-inflammatory cytokine-miRNA network that suppressed 19 pro-inflammatory and pro-fibrotic molecules and increased cardio-reparative molecules (AT2R, EPO and Notch1) as shown in Figure 7. While the change in expression of each individual molecule was modest in response to NP-6A4 treatment in ZO rat heart, the coordinated change in their expression that results in an anti-inflammatory network underlies the powerful anti-inflammatory mode of action of NP-6A4. This is the key mechanism that causes the observed multifactorial improvement in cardiac structure and function (attenuation of hypertrophy and fibrosis, improvement of GLS, GCS, E/e’, MPI and cardiac capillary density) in response to NP-6A4 treatment in ZO rats. To our knowledge, this is the first report that defines an AT2R-induced intracardiac anti-inflammatory cytokine-miRNA network (Figure 7) with cardiovascular and renal protective effects (as predicted by IPA). Suppression of tubulointerstitial injury marker urinary β-NAG in ZO rats by NP-6A4-AT2R signaling is nephroprotective and is in agreement with the IPA prediction that the anti-inflammatory cytokine network activated by NP-6A4 can potentially suppress kidney damage (Figure 4B).

Additional studies are needed to fully understand the role NP-6A4-mediated suppression of ciliary neurotrophic factor (CNTF) that usually exhibit protective effects on the heart. This is also true for Prolactin receptor and TCK1 (Creatine kinase, testis isozyme), two other molecules suppressed by NP-6A4 treatment since their roles in cardiac functions in conditions of untreated obesity and pre-diabetes are not known. Additional studies on NP-6A4-induced structural and functional changes in kidney are also needed to fully understand the impact of NP-6A4 treatment on the pre-diabetic kidney.

We reported previously that NP-6A4 increases cellular respiration and attenuates doxorubicin-induced increase in reactive oxygen species in human VSMCs and protects them from acute serum starvation better than four beta-adrenergic receptor blockers and an AT1R blocker (Mahmood and Pulakat, 2015; Toedebusch et al., 2018). Moreover, NP-6A4-mediated increase in AT2R expression was observed in human VSMCs, and coronary artery and umbilical vein endothelial cells (Toedebusch et al., 2018). Thus, NP-6A4-AT2R signaling renders protective effects on human cardiovascular cells and improves endothelial function by increasing expression of eNOS and nitric oxide. Data presented here show that NP-6A4-induced cardiovascular protective effects also occur in a pre-clinical model for untreated obesity, pre-diabetes, and cardiac dysfunction. Based on our collective *in vitro* and *in vivo* data, we propose that NP-6A4 is a novel therapeutic that can be used alone or synergistic with existing therapeutics (e.g., statins, anti-hyperglycemics and antihypertensives) to treat heart disease presented with preserved ejection fraction, fibrosis and microvascular damage in conditions of obesity and pre-diabetes. It is important to note that patients with obesity and pre-diabetes have an underlying chronic inflammation that makes them highly vulnerable to infectious diseases such as COVID-19, and high morbidity and mortality is seen in both young and old patients with these conditions. It is also noteworthy that while NP-6A4 protected the heart in a pre-clinical model with severe obesity and pre-diabetes (ZO rat), it did not increase expression levels of ACE2, the receptor for SARS-CoV2 in this model. This makes NP-6A4 an ideal adjuvant drug candidate that can provide cardioprotection and mitigate complications arising from severe and/or chronic inflammation in obese and pre-diabetic patients and in pathologies in which increase in ACE2 levels in response to drug treatment is undesirable.

## Data Availability

The original contributions presented in the study are included in the article/Supplementary Material, further inquiries can be directed to the corresponding author.
