# Supplementary material for: Suppression of Inflammatory Cardiac Cytokine Network in Rats with Untreated Obesity and Pre-Diabetes by AT2 Receptor Agonist NP-6A4
Source: Front Pharmacol. 2021 Jun 18;12:693167. doi: 10.3389/fphar.2021.693167 (PMC8253363; doi:10.3389/fphar.2021.693167)
Supplement: Supplementary file 1 [file DataSheet1.DOCX]

**SUPPLEMENTAL INFORMATION**

**Supplemental Table 1: Body weight and fasting plasma chemistry of 10-week old male Zucker lean (ZL) and Zucker obese (ZO) rats shows significant insulin resistance in ZO rats (mean ± SEM)**

| Phenotype | 10-week ZL | 10-week ZO |
| --- | --- | --- |
| Body weight (grams) | 253±8.6 (n=7) | 343±9.1 (n=7) |
| Fasting Insulin (pmol/L) | 115.3±8.4 (n=7) | 1023.9±148.9 (n=7) |
| Fasting Glucose (mmol/L) | 5.62±0.46 (n=7) | 6.37±0.23 (n=7) |
| HOMA-IR | 4.41±0.34 (n=7) | 41.86±5.98 (n=7) |
| Fasting Triglycerides | 0.99±0.07 (n=7) | 3.80±0.69 (n=6) |

**Supplemental Table 2.** **Cardiac parameters of 13-week old male ZO rats treated with either saline or NP-6A4.**

| Cardiac Parameter | ZO+ Saline (n=5) | ZO+NP-6A4 (n= 6) |
| --- | --- | --- |
| Heart Rate | 371±23 | 397±14 |
| Diastolic Blood Pressure (mmHg) | 73±7 | 86±5 |
| Systolic Blood Pressure (mmHg) | 117±6.5 | 128±6 |
| Mean Arterial Pressure (mmHg) | 87±7 | 99±5 |
| Fractional Shortening | 57.6±3.1 | 61.92±1.5 |
| Ejection Fraction | 85.6 ±2.5 | 89.4±1.0 |
| LV Stroke Volume (µL) | 280.75±33.4 | 243.4±37.11 |
| E/e’ | **-31.94±3.24** | **-23.4±1** |
| Myocardial Performance Index (MPI) | **0.52±0.08** | **0.39±0.05** |
| Endo PLAX longitudinal strain (Pk %) | **-14.98±2.21** | **-23.23±2.66** |
| Endo PLAX longitudinal strain rate (Pk (1/s)) | **-3.66±0.64** | **-6.11±0.88** |
| Endo SAX circumferential strain (Pk %) | **-27.64±2.09** | **-34.12±1.54** |
| Endo SAX circumferential strain rate (Pk (1/s)) | **-7.52±0.54** | **-9.49±0.48** |
| Endo SAX radial strain (Pk %) | 25.64±3.14 | 28.15±4.75 |
| Endo SAX radial strain rate (Pk (1/s)) | 4.65±0.7 | 5.25±0.52 |

Values of cardiac parameters that are significantly different (p<0.05) between saline- and NP-6A4-treated rats are shown in bold.

**Supplemental Table 3. Statistically significant differentially expressed intracardiac cytokines in NP-6A4-treated ZO rat heart and used as input for ingenuity pathway analysis.**

| Cytokine | ZO Saline | ZO Saline | ZO Saline | ZO Saline | ZO Saline | ZO  NP-6A4 | ZO  NP-6A4 | ZO  NP-6A4 | ZO  NP-6A4 | ZO  NP-6A4 | Fold change |
| --- | --- | --- | --- | --- | --- | --- | --- | --- | --- | --- | --- |
| EPO | *24.0* | *15.3* | *21.0* | 37.9 | 27.8 | 32.1 | 31.6 | 41.7 | 59.5 | 44.1 | 1.657 |
| Notch-1 | *151.5* | *131.4* | *139.3* | 208.8 | 162.8 | 182.4 | 314.3 | 192.2 | 283.9 | 311.5 | 1.617 |
| GM-CSF | *586.7* | 700.0 | 851.9 | 838.3 | 735.1 | *459.8* | *549.9* | *493.2* | *575.1* | *392.3* | -1.502 |
| IL-1α | *801.1* | 934.2 | 934.4 | 975.2 | *762.5* | *680.8* | *515.9* | *469.8* | *590.1* | *664.1* | -1.508 |
| IL-6 | *1,491.6* | *1,342.1* | *1,280.8* | 2,001.4 | 2,069.8 | *1,150.5* | *1,485.8* | *978.8* | *923.3* | *843.7* | -1.520 |
| TNFα | *3,628.6* | *3,577.0* | 4,790.1 | 4,200.9 | 4,072.7 | *2,391.1* | *2,979.3* | *2,431.6* | *2,658.3* | *2,451.3* | -1.569 |
| ICAM-1 | *3,549.9* | 4,874.8 | 5,160.0 | 5,158.5 | *2,769.1* | 3,799.0 | *2,136.7* | *2,447.3* | *2,322.9* | *2,494.5* | -1.629 |
| L-Selectin | *188.6* | *172.8* | 223.7 | 240.2 | *178.7* | *142.3* | *135.8* | *109.0* | *91.5* | *136.3* | -1.632 |
| PDGF-AA | *620.4* | *381.2* | *509.9* | *412.1* | *503.4* | *232.9* | *238.5* | *244.1* | *300.8* | *457.8* | -1.646 |
| TIMP-1 | *332.6* | 523.8 | 471.9 | 467.7 | *317.9* | *291.2* | *282.8* | *212.1* | *222.6* | *247.6* | -1.682 |
| IL-2 | *3,193.0* | 3,667.5 | 3,664.3 | 4,684.2 | *3,045.2* | *2,065.7* | *2,215.9* | *1,940.5* | *2,102.2* | *2,390.1* | -1.703 |
| Galectin-3 | *579.3* | *402.0* | *532.5* | *247.2* | *349.7* | *317.1* | *153.5* | *148.6* | *197.7* | *390.2* | -1.748 |
| IL-13 | *1,535.3* | 1,711.7 | *1,443.4* | 1,766.9 | 1,576.2 | *886.5* | *1,063.5* | *503.4* | *986.7* | *1,062.9* | -1.784 |
| Prolactin R | *2,486.2* | *1,616.5* | 2,960.2 | 3,127.7 | *2,375.7* | *1,099.2* | *1,015.2* | *1,049.7* | *1,149.4* | *1,752.0* | -2.071 |

**Supplemental Table 4a. MicroRNAs that exhibited increased expression in NP-6A4 treated ZO rat heart**

| MicroRNA | fold change | P value |
| --- | --- | --- |
| rno-miR-7a-1-3p | 4.302078351 | 0.000352 |
| rno-miR-361-3p | 2.502402051 | 0.006869 |
| rno-miR-509-5p | 2.286127394 | 0.014174 |
| rno-miR-148b-3p | 2.264436055 | 0.014668 |
| rno-miR-190a-3p | 2.238872141 | 0.009123 |
| rno-miR-34b-3p | 2.222313884 | 0.011015 |
| rno-miR-101b-3p | 2.18687779 | 0.008948 |
| rno-miR-203a-3p | 2.08374769 | 0.024259 |
| rno-miR-1839-3p | 2.02679644 | 0.001335 |
| rno-miR-322-5p | 1.9511753 | 0.005697 |
| rno-miR-133a-5p | 1.940915352 | 0.000735 |
| rno-let-7f-5p | 1.879978548 | 0.017714 |
| rno-miR-10a-5p | 1.848737509 | 0.010179 |
| rno-miR-30e-3p | 1.837270947 | 0.022885 |
| rno-miR-362-3p | 1.826530393 | 0.021701 |
| rno-miR-15b-5p | 1.778807509 | 0.000178 |
| rno-miR-493-3p | 1.766137664 | 0.0165 |
| rno-miR-3584-5p | 1.751497829 | 0.004434 |
| rno-miR-224-5p | 1.703552253 | 0.036249 |
| rno-miR-25-3p | 1.691181148 | 0.006105 |
| rno-miR-146a-5p | 1.688015323 | 0.000229 |
| rno-miR-199a-3p | 1.6757675 | 0.010313 |
| rno-miR-338-5p | 1.663895006 | 0.000575 |
| rno-miR-30b-5p | 1.663347959 | 0.00733 |
| rno-miR-128-3p | 1.626907921 | 0.037964 |
| rno-miR-200c-3p | 1.601692945 | 0.000128 |
| rno-miR-19b-3p | 1.579759783 | 0.027128 |
| rno-miR-221-5p | 1.57720945 | 0.020319 |
| rno-miR-1-3p | 1.55164659 | 0.014609 |
| rno-miR-350 | 1.548995765 | 0.030471 |

**Supplemental Table 4b. MicroRNAs that were suppressed in NP-6A4 treated ZO rat heart.**

| MicroRNA | fold change | P Value |
| --- | --- | --- |
| rno-miR-324-3p | -1.504196244 | 0.004529 |
| rno-miR-339-5p | -1.504803386 | 0.002412 |
| rno-miR-874-3p | -1.525491139 | 0.00289 |
| rno-miR-23b-5p | -1.529757529 | 0.011892 |
| rno-miR-6324 | -1.546012518 | 0.001011 |
| rno-miR-342-5p | -1.547909581 | 0.001211 |
| rno-miR-210-5p | -1.55066323 | 0.006056 |
| rno-miR-339-3p | -1.570515521 | 0.00281 |
| rno-miR-500-3p | -1.578078968 | 0.000102 |
| rno-miR-672-5p | -1.586122061 | 0.036659 |
| rno-miR-181c-3p | -1.598822522 | 0.005505 |
| rno-miR-351-5p | -1.601993174 | 0.000632 |
| rno-miR-125a-3p | -1.614086841 | 8.63E-05 |
| rno-miR-379-5p | -1.630968139 | 0.017453 |
| rno-miR-224-3p | -1.653018189 | 0.015858 |
| rno-miR-674-5p | -1.654505398 | 0.002557 |
| rno-miR-425-3p | -1.655607857 | 0.001935 |
| rno-miR-490-5p | -1.673983607 | 0.006601 |
| rno-miR-542-5p | -1.721880952 | 1.83E-05 |
| rno-miR-187-3p | -1.724273227 | 0.010747 |
| rno-miR-664-2-5p | -1.740147743 | 0.003816 |
| rno-miR-503-5p | -1.77683387 | 0.000352 |
| rno-mir-124-3 | -1.802138386 | 0.040592 |
| rno-mir-124-1 | -1.802138386 | 0.040592 |
| rno-mir-124-2 | -1.802138386 | 0.040592 |
| rno-miR-210-3p | -1.819545842 | 0.000547 |
| rno-miR-505-5p | -1.820552064 | 0.00197 |
| rno-miR-193-5p | -1.82177375 | 0.00034 |
| rno-miR-320-3p | -1.824746433 | 0.000129 |
| rno-miR-93-3p | -1.825134266 | 0.00036 |
| rno-miR-296-3p | -1.832176895 | 0.019892 |
| rno-miR-423-3p | -1.833273714 | 2.8E-05 |
| rno-miR-667-3p | -1.835314368 | 0.011898 |
| rno-miR-139-3p | -1.835594259 | 0.000124 |
| rno-miR-760-3p | -1.864139983 | 0.018872 |
| rno-miR-99b-3p | -1.918032545 | 4.6E-05 |
| rno-miR-490-3p | -1.925312726 | 0.000745 |
| rno-miR-150-3p | -1.96128528 | 0.018254 |
| rno-miR-23a-5p | -1.996787768 | 0.005129 |
| rno-miR-541-5p | -1.998086445 | 0.005094 |
| rno-miR-124-3p | -2.004146816 | 0.032366 |
| rno-miR-212-3p | -2.042643734 | 0.007536 |
| rno-miR-217-5p | -2.054017965 | 0.007902 |
| rno-miR-25-5p | -2.059712249 | 0.000124 |
| rno-miR-326-3p | -2.075873022 | 8.77E-05 |
| rno-miR-351-3p | -2.098766693 | 0.000122 |
| rno-miR-330-3p | -2.111205187 | 5.99E-05 |
| rno-miR-127-3p | -2.116098818 | 0.002065 |
| rno-miR-214-3p | -2.229060847 | 2.83E-05 |
| rno-miR-138-5p | -2.239093849 | 0.000851 |
| rno-miR-877 | -2.264843558 | 0.001697 |
| rno-miR-204-3p | -2.350621738 | 0.01235 |
| rno-miR-423-5p | -2.399455738 | 6.7E-05 |
| rno-miR-433-3p | -2.401173083 | 0.000965 |
| rno-miR-134-5p | -2.406695467 | 0.004933 |
| rno-miR-182 | -2.438836427 | 0.003711 |
| rno-miR-298-5p | -2.507224876 | 2.47E-05 |
| rno-miR-485-5p | -2.535942792 | 0.002349 |
| rno-miR-337-5p | -3.455299103 | 0.031568 |
| rno-miR-665 | -3.574984033 | 0.001254 |
| rno-miR-3099 | -3.70343445 | 0.028961 |

**Supplemental Table 5: Potential predicted relationship between NP-6A4-suppressed intracardiac microRNAs and NP-6A4-induced cytokines based on the 3’ untranslated sequences of human mRNAs encoding these cytokines as input for RegRNA software.**

| Intracardiac Protein:  Fold change | mRNA:NCBI Ref Seq and length | Coding sequence | miRNA/fold change | Binding Location | Binding site sequence |
| --- | --- | --- | --- | --- | --- |
| AT2R/+1.5 | NM_000686.4  1-2906 | 208-1299 | miR-330-3p/-2.11 | 2060-2082 | miRNA: 3' agAGACGUCCGGCAC-ACGAAACg 5'  \|:\| :\| \| :\|\|\| \|\|\|\|\|\|\|  Target:5' acTTTTTA-GATGTGCTGCTTTGa 3' |
|  |  |  | miR-138-5p/-2.24 | 2532-2552 | miRNA: 3' gccGGACUAAGUGUUGUGGUCGa 5'  \|:\| \|\|\| \| ::\|\|\|\|\|\|  Target:5' agaCTTTATT--CTGTACCAGCc 3' |
| NOTCH1/+1.62 | NM_017617.4  1-9322 | 1-7668 | miR-217-5p/-2.05 | 7090-7111 | miRNA: 3' agGUUAGUCAAGGACUACGUCau 5'  \|\|\| \| \|\| \| \|\|\|\|\|\|\|  Target:5' caCAA-AACTTACAGATGCAGca 3' |
|  |  |  |  | 8322-8348 | miRNA: 3' agguuAGUCAAGG----ACUACGUCAu 5'  \|\|\|\| \|:\| \| \|\|\|:\|\|\|  Target:5' ttgttTCAGGTTCAGTATTATGTAGTt 3' |
|  |  |  | miR-330-3p/-2.11 | 8623-8646 | miRNA: 3' agaGACGUCCG-GCACACGAAACg 5'  \| \|::\|\|\| \| \|\|\|:\|\|\|\|  Target:5' cccCCGTGGGCTCCCGTGTTTTGt 3' |
|  |  |  |  | 8686-8706 | miRNA: 3' agaGACGUCCGGCACACGAAACg 5'  \| \|::\|\|\|:\| \|\| \|\|\|\|\|  Target:5' acgCCGTGGGCTGCGTCCTTTGg 3' |
|  |  |  | miR-138-5p/-2.24 | 8079-8100 | miRNA: 3' gcCGGACUAAGUGUUGUGGUCGa 5'  \|\|\| \|: \|:\| \|\|\|\|\|\|\|  Target:5' caGCCCGG-CCGCTCCACCAGCa 3' |
|  |  |  | miR-423-5p | 8358-8380 | miRNA: 3' uuucagagCGAGAGACGGGGAGu 5'  \|:\|\|\|: \|:\|\|\|\|\|  Target:5' ttatacaaGTTCTTGGTCCCTCc 3' |
|  |  |  | miR-433-3p | 8296-8318 | miRNA: 3' ugUGGCU-CCUCGGGUAGUACUa 5'  \|\|\| \| \|\| \|\| \|\|\|\|\|\|\|  Target:5' gcACCCATGGTACCAATCATGAa 3' |
|  |  |  | miR-182/-2.43 | 8207-8229 | miRNA: 3' ucACACUCAAGAUGGUAACGGUuu 5'  \| \|\|:\|\|\| \| ::\|\|\|\|\|\|  Target:5' caTATGGGTTAGA-TGTTGCCAtg 3' |
|  |  |  | miR-665 | 8015-8033 | miRNA: 3' uccccggagUCGGAGGACCa 5'  \|\| \|\|\|\|\|\|\|  Target:5' agtccaaaaAG-CTCCTGGg 3' |
| EPO/+1.66 | NM_000799.3  1-1340 | 182-763 | miR-138-5p/-2.24 | 784-806 | miRNA: 3' gcCG-GACUAAGUGUUGUGGUCGa 5'  \|\| \|\| \| \| \|\|:\|:\|\|\|\|\|  Target:5' ggGCTCTCA-GCTCAGCGCCAGCc 3' |
|  |  |  | miR-182/  -2.43 | 1217-1245 | miRNA: 3' ucACACUCAAGA---UG--GUAACGGUUu 5'  \|\|\|\| \|\|\|\| \|\| \|\|\|\|\| \|\|\|  Target:5' ttTGTGTATTCTTCAACCTCATTGACAAg 3' |
|  |  |  | miR-485-5p | 786-809 | miRNA: 3' cuuaAGU--AGUGCCGGUCGGAga 5'  \|\|\| \|\|\| \|\|\|\|\|\|\|\|  Target:5' gctcTCAGCTCAGCGCCAGCCTgt 3' |
| EPHA5/+2.3 | NM_004439.7  1-8435 | 754-3867 | miR-217-5p/-2.05 | 4770-4791 | miRNA: 3' agguUAGUCAAGGACUACGUCAu 5'  \|\|\|\|\|\| \| \|\|\|\|\|\|\|  Target:5' tgatATCAGTGAC-AATGCAGTt 3' |
|  |  |  |  | 5302-5329 | miRNA: 3' agguUAGUCA--AGG---ACUACGUCAu 5'  \|\|: \|\| \| \| \| \|\|\|\|\|\|\|  Target:5' ttatATTTGTGATACAAATTATGCAGTg 3' |
|  |  |  | miR-877/  -2.26 | 7212-7234 | miRNA: 3' gggACGC---GGUAGAGGAGAUg 5'  \|\|:\| :\|\|\| \|\|:\|\|\|\|  Target:5' ttaTGTGTTTTCATGTCTTCTAc 3' |
|  |  |  | miR-182/  -2.43 | 7472-7499 | miRNA: 3' ucACAC--UCA--AGAUGGUAACGGUUu 5'  \|\|\|\| \|\|\| \|\|::\| \|\|\|\|:\|\|  Target:5' agTGTGATAGTAAACTGTCTTTGCTAAt 3' |
|  |  |  |  | 4833-4862 | miRNA: 3' ucACACUCAAGA----UGGU--AACGGUUu 5'  \| \|\|\|\|\| \|\| \| :\| \|\|\|:\|\|\|  Target:5' ttTCTGAGTCCTGAAAAGTATCTTGTCAAg 3' |
|  |  |  | miR-298-5p/-2.50 | 4903-4929 | miRNA: 3' acCCU--CUUGG-AGGGACGAAGACGa 5'  \|\|\| \|\|\|: \| \| \|\|\|\|\|\|\|:  Target:5' aaGGATTTAACTGTGCAGGCTTCTGTt 3' |
|  |  |  | miR-337-5p/-3.45 | 7654-7674 | miRNA: 3' uuGAGGACAUACU-UCGGCAAg 5'  \|\| \|\|\|\|\| \| \|\|\|:\|\|\|  Target:5' aaCT-CTGTAGCACAGCTGTTt 3' |

**Supplemental Fig. 1. IPA-predicted Diseases and Functions networks suppressed by NP-6A4 in ZO rat heart.**

**
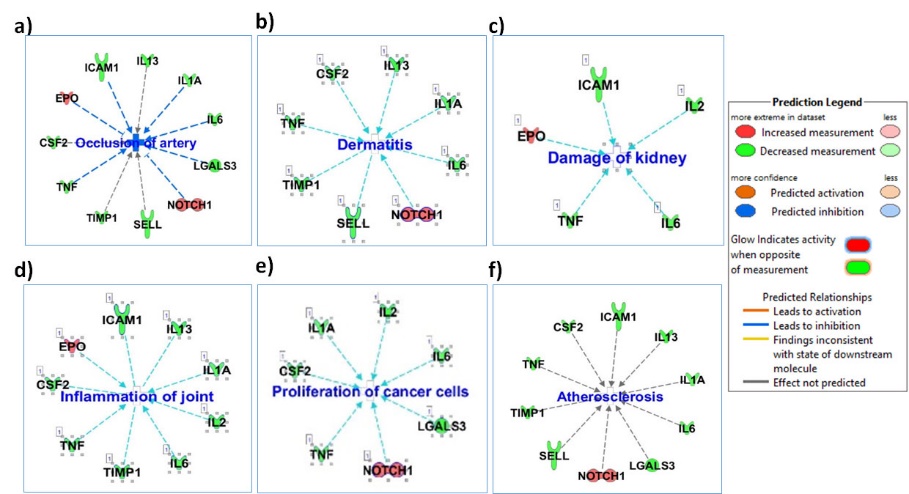
**

Blue color denotes diseases and functions in the ZO rat heart suppressed by NP-6A4 treatment according to IPA predictions. **a**) Occlusion of Artery: activation Z score = -2.646; p value 1.39E-13; **b**) Atherosclerosis: activation Z score = -2.449; p value 2.61E-12; **c**) Damage to kidney: activation Z score = -2.216; p value 5.35E-12; **d**) Inflammation of Joint: activation Z score = -2.175; p value 1.33E-07; **e**) Proliferation of Cancer Cells: activation Z score = -2.171; p value 1.40E-08; **f**) Dermatitis: activation Z score = -2.070; p value 1.39E-09
